# Ivabradine for the Prevention of Anthracycline-Induced Cardiotoxicity in Female Patients with Primarily Breast Cancer: A Prospective, Randomized, Open-Label Clinical Trial

**DOI:** 10.3390/medicina59122140

**Published:** 2023-12-09

**Authors:** Eglė Čiburienė, Sigita Aidietienė, Greta Ščerbickaitė, Eglė Sadauskienė, Diana Sudavičienė, Edita Baltruškevičienė, Birutė Brasiūnienė, Monika Drobnienė, Jelena Čelutkienė

**Affiliations:** 1 Clinic of Cardiac and Vascular Diseases, Institute of Clinical Medicine, Faculty of Medicine, Vilnius University, 03101 Vilnius, Lithuania; 2 Center of Cardiology and Angiology, Vilnius University Hospital “Santaros Clinics”, 08661 Vilnius, Lithuania; 3 Department of Medical Oncology, National Cancer Institute, 08406 Vilnius, Lithuaniamonika.drobniene@nvi.lt (M.D.); 4 Faculty of Medicine, Vilnius University, 03101 Vilnius, Lithuania

**Keywords:** cardio-oncology, cancer-therapy-induced cardiotoxicity, cardiotoxicity prevention

## Abstract

*Background and Objectives:* Cancer therapy containing anthracyclines is associated with cancer-treatment-related cardiac dysfunction and heart failure (HF). Conventional cardioprotective medications can be frequently complicated by their blood-pressure-lowering effect. Recently, elevated resting heart rate was shown to independently predict mortality in patients with cancer. As a heart rate-lowering drug without affecting blood pressure, ivabradine could present an alternative management of anthracyclines-induced cardiotoxicity. *Materials and Methods:* This study aimed to investigate the probable protective effects of ivabradine in cancer patients with elevated heart rate (>75 beats per minute) undergoing anthracycline chemotherapy. Patients referred by oncologists for baseline cardiovascular risk stratification before anthracycline chemotherapy who met the inclusion criteria and had no exclusion criteria were randomly assigned to one of two strategies: ivabradine 5 mg twice a day (intervention group) or controls. Electrocardiogram, transthoracic echocardiogram with global longitudinal strain (GLS), troponin I (Tn I), and N-terminal natriuretic pro-peptide (NT-proBNP) were performed at baseline, after two and four cycles of chemotherapy and at six months of follow-up. The primary endpoint was the prevention of a >15% reduction in GLS. Secondary endpoints were effects of ivabradine on Tn I, NT-proBNP, left ventricular (LV) systolic and diastolic dysfunction, right ventricle dysfunction, and myocardial work indices. *Results:* A total of 48 patients were enrolled in the study; 21 were randomly assigned to the ivabradine group and 27 to the control group. Reduced GLS was detected 2.9 times less often in patients receiving ivabradine than in the control group, but this change was non-significant (OR [95% CI] = 2.9 [0.544, 16.274], *p* = 0.208). The incidence of troponin I elevation was four times higher in the control group (OR [95% CI] = 4.0 [1.136, 14.085], *p* = 0.031). There was no significant change in NT-proBNP between groups, but the increase in NT-proBNP was almost 12% higher in the control group (OR [95% CI] = 1.117 [0.347, 3.594], *p* = 0.853). LV diastolic dysfunction was found 2.7 times more frequently in the controls (OR [95% CI] = 2.71 [0.49, 15.10], *p* = 0.254). Patients in the ivabradine group were less likely to be diagnosed with mild asymptomatic CTRCD during the study (*p* = 0.045). No differences in right ventricle function were noted. A significant difference was found between the groups in global constructive work and global work index at six months in favour of the ivabradine group (*p* = 0.014 and *p* = 0.025). Ivabradine had no adverse effects on intracardiac conduction, ventricular repolarization, or blood pressure. However, visual side effects (phosphenes) were reported in 14.3% of patients. *Conclusions:* Ivabradine is a safe, well-tolerated drug that has shown possible cardioprotective properties reducing the incidence of mild asymptomatic cancer-therapy-induced cardiac dysfunction, characterised by a new rise in troponin concentrations and diminished myocardial performance in anthracycline-treated women with breast cancer and increased heart rate. However, more extensive multicentre trials are needed to provide more robust evidence.

## 1. Introduction

Anthracyclines (AC) have been the basis of treatment for many solid cancers and haematological malignancies for over 60 years. However, these chemotherapy drugs are the most common reason for chemotherapy-induced cardiotoxicity that limits optimal cancer treatment [1,2]. Therefore, extensive efforts are underway to find the best cardioprotective strategy for anthracycline-induced cardiotoxicity (AIC) [3]. Beta-blockers (BBs) and angiotensin-converting enzyme inhibitors/angiotensin receptor blockers (ACEIs/ARBs) are used to treat chemotherapy-induced cardiotoxicity, but adverse inotropic and blood-pressure-lowering effects limit their use [4,5,6,7,8]. This is particularly important for oncology patients, who often experience hypotension due to weight loss, vomiting, and diarrhoea.

Anker et al. showed that a threshold heart rate of ≥75 beats per minute (BPM) is an independent predictor of mortality in colorectal, pancreatic, and non-small cell lung cancer patients [9]. 

Ivabradine is a heart-rate-lowering drug with no effect on blood pressure that is indicated in patients with symptomatic heart failure and reduced left ventricular systolic function (LVEF) who are in sinus rhythm and have a heart rate ≥75 BPM despite the highest tolerated dose of BBs, or when BBs are contraindicated [10]. Reduction in tachycardia ameliorates oxidative and inflammatory status, endothelial dysfunction, and arterial stiffness, as well as improves myocardial perfusion. These effects can benefit patients with chronic cardiovascular, pulmonary, and renal disease and cancer [11]. Moreover, it was proved that ivabradine reduced cardiovascular deaths and hospitalizations in patients with HF and elevated heart rate [12]. 

Most data on the cardioprotective effects of ivabradine against doxorubicin-induced cardiomyopathy are based on experimental studies in rats. In these studies, ivabradine demonstrates an anti-remodelling effect by multiple mechanisms, including antifibrotic, anti-inflammatory, antioxidant, and anti-apoptotic effects [13,14]. 

A 2017 study by Vasyuk et al. investigated the cardioprotective effects of ivabradine in a randomized study enrolling women with breast cancer treated with doxorubicin. It showed that ivabradine was safe, did not cause bradycardia, reduced patients’ palpitations, and helped preserve normal left ventricular global longitudinal deformation. Cardiac biomarkers were not evaluated in this study [15].

We hypothesized that ivabradine might protect cancer patients undergoing chemotherapy containing anthracyclines from AIC and HF. Therefore, we conducted this prospective, randomized, open-label, single-centre clinical trial to investigate the protective effects of ivabradine in adult cancer patients undergoing anthracycline-based chemotherapy.

## 2. Materials and Methods

### 2.1. Study Design

The trial was a prospective, randomized, open-label study conducted at the Vilnius University hospital “Santaros Clinics”, a Cardiology and Angiology clinic (Vilnius, Lithuania). Patients were referred from the National Cancer Institute.

This study complied with the Declaration of Helsinki. The study protocol was approved by the State Medicines Control Agency and Lithuanian Bioethics Committee (protocol code ICO, 2019-04-16, No. P-19-12), and was registered on ClinicalTrials.gov on 24/07/2019 (No. NCT04030546) and on EudraCT (No. 2019-000661-20).

All participants provided written informed consent.

### 2.2. Study Participants

A total of 48 women with solid tumours, specifically breast cancer (46) and sarcoma (2), scheduled for anthracycline therapy and possessing an elevated heart rate (>75 BPM) at Vilnius University hospital “Santaros Clinics” between May 2019 and January 2022 were in included in the study.

Medical oncologists referred patients to the cardiologist before anthracycline-based chemotherapy for baseline cardiovascular (CV) risk stratification [16].

Eligibility requirements included an age of at least 18 years, planned chemotherapy with AC, resting HR >75 BPM on electrocardiogram (ECG), and blood pressure measurements. Before the ECG, patients rested for 10 min. After the ECG, the heart rate was obtained twice with a 5 min break to measure vital signs. The study only included patients whose heart rate was >75 BPM on all three measurements from the ECG and blood pressure manometer. 

Exclusion criteria included contraindications for ivabradine administration; concomitant use of angiotensin-converting enzyme inhibitor (ACEI) or angiotensin receptor blocker (ARB) and beta-blocker (BB); chronic kidney disease (GFR < 30 mL/min.); baseline LVEF < 50%; inability to complete informed consent; severe valve disease; other severe conditions; and poor echogenicity. 

Patients with AF were excluded from the study as a contraindication to ivabradine.

### 2.3. Randomization, Allocation, and Intervention

Randomization was on a 1:1 ratio to receive ivabradine or standard care (the observation during cardiotoxic treatment with anthracyclines). No placebo was used in this study. The randomization was performed by a computer-based randomization list generated using GraphPad by Dotmatics Prism program. Randomization and allocation data were maintained by an independent research coordinator. A blinded investigator performed all echocardiograms.

The initial dose of ivabradine was 5 mg twice daily, subsequently adjusted according to the heart rate. Ivabradine was continued until completion of anthracycline chemotherapy.

### 2.4. Study Procedures

All study patients underwent an ECG, transthoracic echocardiogram (TTE), and routine laboratory analyses including cardiac-specific biomarkers (high-sensitivity cardiac Tn I and NT-proBNP) and a biochemistry panel [17,18].

The following sequential ECG, TTE, Tn I, and NT-proBNP were performed after the second and the fourth cycle of anthracycline chemotherapy and at six months of follow-up.

All TTEs were performed by a single experienced cardiologist using the same Vivid E95™ GE Healthcare system to estimate LVEF and volumes, LV diastolic function, GLS and global myocardial work (MW) parameters, valves’ assessment, and RV function. Simpson’s 2D and semi-automated 3D assessment of LVEF were performed. Diastolic LV dysfunction was identified when more than half of the variables listed below were abnormal: septal e’ < 7 cm/s, lateral e’ < 10 cm/s, average E/e’ ratio > 14, left atrium volume index (LAVI) > 34 mL/m^2^, and peak tricuspid regurgitation (TR) velocity > 2.8 m/s. RV dysfunction was determined by tricuspid annular plane systolic excursion (TAPSE) < 1.7 cm and/or tricuspid annular systolic velocity by tissue Doppler (S’) < 9.5 cm/s [19].

MW parameters, which included global work index (GWI), global constructive work (GCW), global wasted work (GWW), and global work efficiency (GWE), were assessed by the 2D strain–pressure loop using software version 204 for offline data analysis in an Echo PAC GE Healthcare workstation. GWI < 1310 mmHg%, GCW < 1543 mmHg%, GWW > 287 mmHg%, and GWE < 90% were defined as abnormal [20].

According to the laboratory parameters, Tn I > 99th percentile and NT-proBNP ≥ 125 pg/mL or a new significant (>20%) increase from the baseline were considered elevated. Troponin I concentrations were measured using Abbott Diagnostics (Germany) reagents on an Architect systems analyser: elevated Tn I concentration is >35 ng/L for men and >16 ng/L for women.

Arterial hypertension was diagnosed in patients with high CV risk when blood pressure exceeded ≥ 130 mmHg systolic and/or ≥80 mmHg diastolic, and in other patients if blood pressure exceeded ≥ 140 mmHg systolic and/or ≥90 mmHg diastolic [21].

Dyslipidaemia was diagnosed according to 2019 ESC guidelines for the management of dyslipidaemia [22]. 

Mild asymptomatic cancer-therapy-related cardiac dysfunction (CTRCD) was defined according to the latest 2022 European Society of Cardiology (ESC) guidelines on cardio-oncology as LVEF > 50% and new relative decrease in GLS by >15% from the baseline and/or a new increase in cardiac biomarkers 

After randomization, ivabradine was initiated on the first day of chemotherapy.

At each visit (except the screening visit), ivabradine consumption was monitored by counting the number of tablets consumed based on the patient’s returned packages. The independent research coordinator was responsible for recording the study drug intake. The recommended minimum amount of ivabradine consumed to ensure the reliability of the study data was more than 80% of the total dose, the maximum possible duration of ivabradine administration. 

### 2.5. Study Endpoints

The primary endpoint of this study was the incidence of LV dysfunction as measured by a relative decline in the GLS of >15% from the baseline value.

Secondary endpoints of the trial:

1. Incidence of myocardial damage as measured by elevated high-sensitivity cardiac Tn I.
2. Incidence of myocardial injury according to elevated NT-proBNP levels.
3. Incidence of LV diastolic dysfunction.
4. Incidence of LV systolic dysfunction measured by EF and symptomatic HF.
5. Incidence of RV dysfunction.
6. Changes in myocardial work parameters by the 2D strain–pressure loop.
7. Incidence of adverse effects of ivabradine.

**Figure 1:**
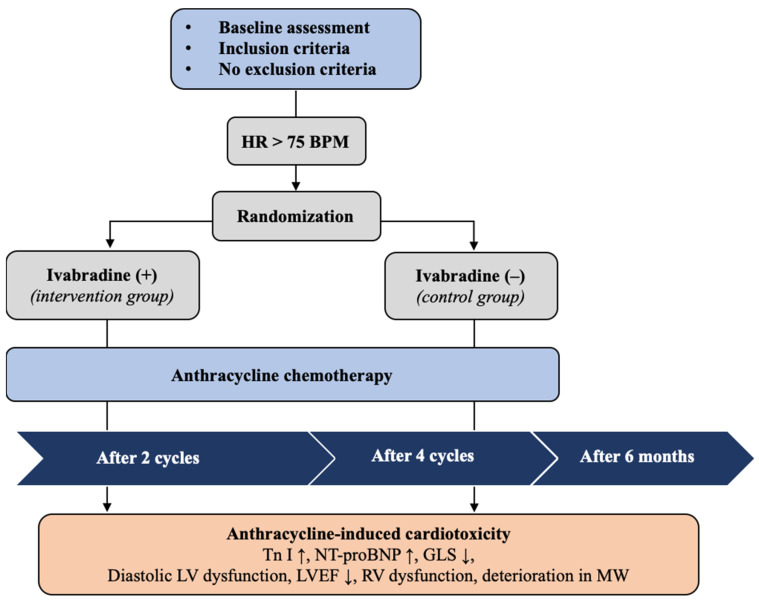
Protocol scheme. GLS—global longitudinal strain; HR—heart rate; LVEF—left ventricular ejection fraction; MW—myocardial work; NT-proBNP—N-terminal natriuretic pro-peptide; RV—right ventricle; Tn I—troponin I.

The protocol scheme is depicted in Figure 1.

### 2.6. Statistical Analysis

The sample size was calculated with an expected incidence of GLS reduction of 20% with the use of AC and an expected reduction to 10% with the addition of ivabradine [23,24]. Assuming a superiority margin of 20% (i.e., δ = 0.2), the actual mean outcome rates of treatment and active control are 10% (pT = 0.1) and 20% (pC = 0.2), respectively, to achieve 80% power (1-β = 0.8) at the 5% significance level (α = 0.05) with equal allocation (k = 1) and a dropout rate of 0%, and the total sample size is 36: 18 for treatment and 18 for active control [25].

Descriptive statistics such as frequency tables and medians (Q1–Q3) were used to describe quantitative and qualitative data. The Kolmogorov–Smirnov test was used to assess the normality of the data. Most parameters were non-normally distributed, so non-parametric statistical analyses were chosen. The Fisher exact test assessed differences between treatment groups and categorical clinical parameters. The Mann–Whitney U test evaluated differences between treatment groups and continuous parameters. The Wilcoxon signed-rank test was used to compare baseline clinical parameter values with the same parameter values at different time points. The univariate logistic regression model was used to evaluate the odds ratio of ivabradine administration. A two-tailed *p*-value less than 0.05 was considered significant. Statistical analysis was performed using the Statistical Analysis System (SAS) package version 9.2.

## 3. Results

From May 2019 to January 2022, we screened 120 patients who were referred for cardiotoxic risk assessment before planned anthracycline treatment. All were females with a median age of 48 years. 

A total of 66 patients were not eligible for the study, for the following reasons: 36 patients had resting HR < 75 BPM; 19 patients were using ACEIs/ARBs or BBs; 11 had poor echogenicity. Furthermore, five patients refused to participate in the study.

We randomised the remaining 48 patients. A total of 21 patients were randomly assigned to the ivabradine group and 27 patients to the control group. 

**Figure 2:**
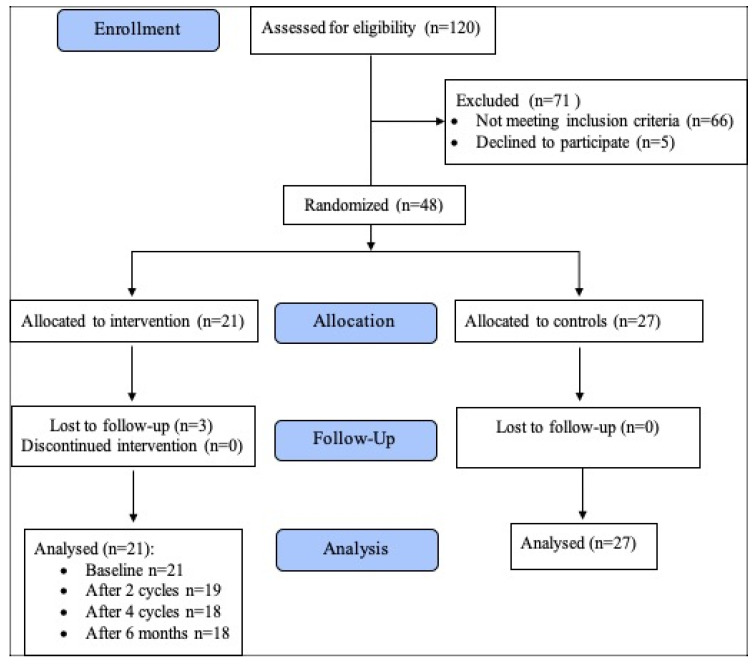
Clinical trial diagram.

During the study, three ivabradine group patients were lost to follow-up. The follow-up was completed in August 2022. The diagram of the study is presented in Figure 2. 

Breast cancer was diagnosed in 46 patients (20 on the right side, 25 on the left side, and 1 had bilateral breast cancer); 2 patients presented with sarcoma. The main anthracyclines prescribed were doxorubicin and epirubicin, with median cumulative doses of 242 (180–375) mg/m^2^ and 360 (360–360) mg/m^2^, respectively. The median number of cycles of anthracyclines was 4 (3–5), delivered over 65 (63–73) days. 

The baseline characteristics of the patients were statistically balanced between the groups. Patients’ baseline characteristics are shown in Table 1. 

### 3.1. Cardiovascular Toxicity Risk Stratification before Anticancer Therapy

Cardiovascular toxicity risk assessment was accomplished for every patient, and modification of the risk factors was suggested, including the recommendation for moderate physical activity during cancer treatment. 

Most patients (83%) were classified as having a low cardiotoxicity risk. However, four patients in the ivabradine group and three in the control group were at intermediate risk (due to age > 65 years and multiple CV risk factors), and one patient in the control group was at high risk of cardiotoxicity (because of previous anthracycline use).

### 3.2. Ivabradine Dosage and Efficacy

The starting dose of ivabradine was 5 mg twice daily. The dose of ivabradine was adjusted at subsequent visits according to the heart rate and side effects. If the heart rate was >75 BPM, the dose was increased to a maximum dose of 7.5 mg twice daily. In four patients, ivabradine was increased to 7.5 mg twice a day. In three patients with heart rates > 75 BPM, the ivabradine dose was not increased due to visual side effects. No patient had to discontinue ivabradine or reduce the dose due to bradycardia. The ivabradine compliance rate was 98.6%. 

**Figure 3:**
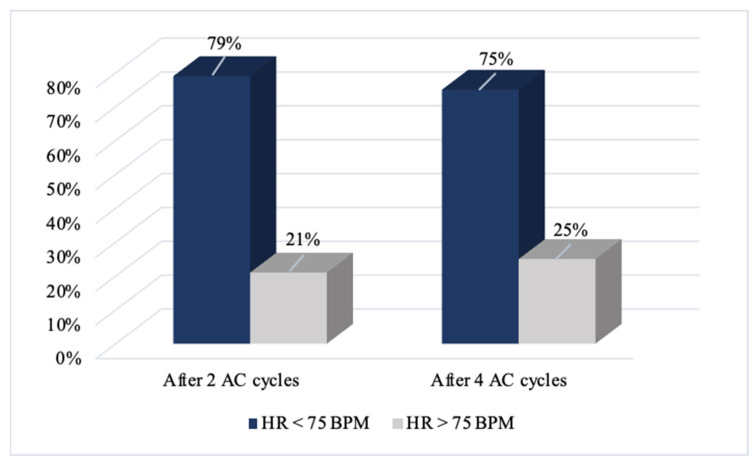
Heart rate achievement during the study in ivabradine group patients. AC—anthracyclines; BPM—beats per minute; HR—heart rate.

The heart rates achieved in ivabradine group patients are presented in Figure 3.

In 79% of patients taking ivabradine, HR decreased by <75 BPM at the visit after two cycles of chemotherapy and remained at 75% after four cycles of anthracyclines.

### 3.3. Primary Endpoint

A new relative decline in GLS by >15% from the baseline was found in nine patients (19%). Two (9.5%) were assigned to ivabradine and seven (26%) to control group (*p* = 0.270). GLS decrease in ivabradine group patients was 2.9 times less than in the control group, but this was not statistically significant (OR [95% CI] = 2.9 [0.544, 16.274], *p* = 0.208).

**Figure 4:**
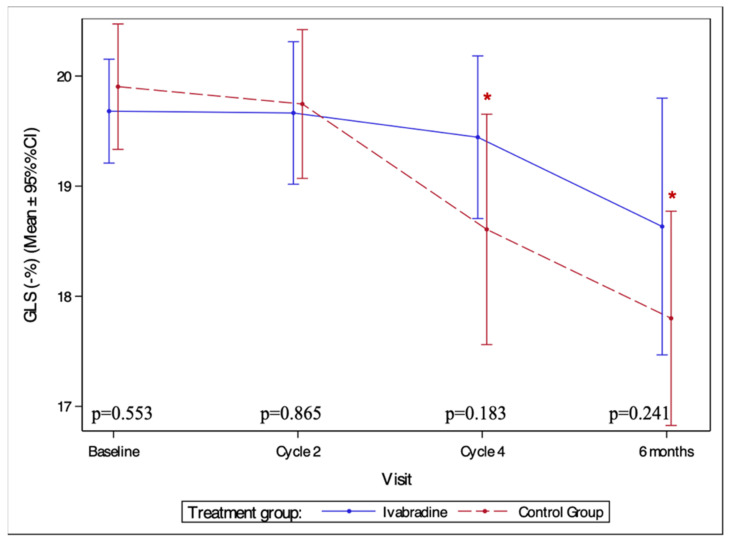
Changes of global longitudinal strain in study groups during the study period. GLS—global longitudinal strain. *—statistically significant difference compared to baseline (*p* < 0.05).

In the ivabradine group, GLS has not significantly changed from the baseline. In contrast, a significant decrease in GLS was observed in the control group after four AC cycles and at 6 months follow-up compared to baseline (*p* = 0.023 and *p* < 0.001, respectively) (Figure 4). 

### 3.4. Secondary Endpoints

#### 3.4.1. Changes in the Levels of Tn I 

During the follow-up, elevation of Tn I was found in 20 patients (42%). Of these patients, five (24%) were in ivabradine and fifteen (56%) in the controls (*p* = 0.04). 

Tn I elevation was observed four times more frequently in the controls than in the ivabradine group patients (OR [95% CI] = 4 [1.136, 14.085], *p* = 0.031).

**Figure 5:**
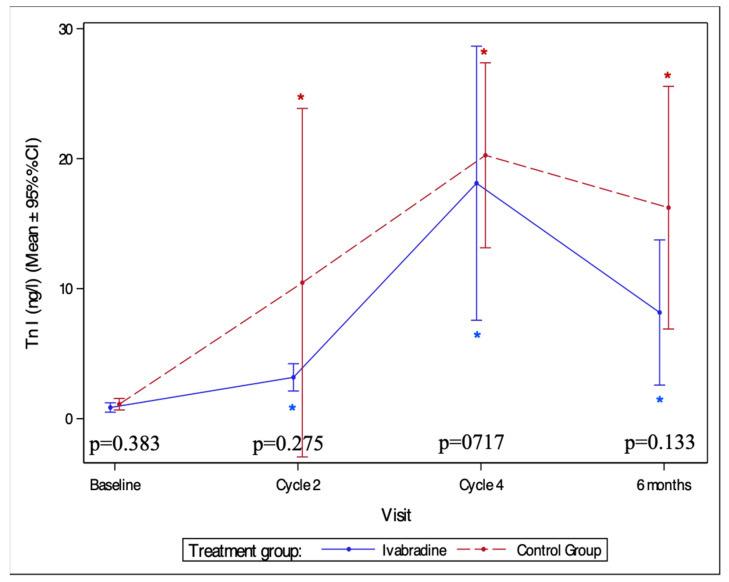
Levels of troponin I during the study period. Tn I—troponin I; *—statistically significant difference compared to baseline (*p* < 0.05).

The most significant increase in median Tn I was observed after four cycles of AC: 10 (6–21) in the ivabradine group (*p* = 0.03) and 14 (9–31) in the controls (*p* < 0.01) (Figure 5). However, there was no statistically significant difference of troponin levels between the groups.

#### 3.4.2. Changes in NT-proBNP Levels

NT-proBNP increased in 19 patients (39.6%). Of these, eight were in ivabradine group and eleven in the controls (*p* = 0.926). The highest rise in median NT-proBNP was seen after four AC cycles: 80 (53–153) in the ivabradine and 84 (44–148) in the control group (*p* = 0.315). 

The increase in NT-proBNP was observed almost 12% more frequently in the control group (OR [95% CI] = 1.117 [0.347, 3.594], *p* = 0.853). However, no statistically significant differences were found between groups during the observation period. 

#### 3.4.3. LV Diastolic Dysfunction

LV diastolic dysfunction evolved in eight (16.7%) patients (two in the ivabradine group and six in the controls, *p* = 0.437). The diastolic dysfunction of LV was found 2.7 times more often in the control group than in ivabradine group patients during the study period, but the difference was not statistically significant (OR [95% CI] = 2.714 [0.488, 15.103], *p* = 0.254).

#### 3.4.4. LV Systolic Dysfunction and Symptomatic HF 

**Figure 6:**
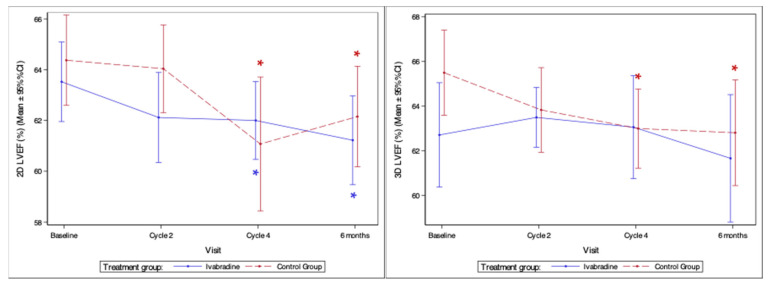
Changes of left ventricular ejection fraction. 2D—two-dimensional; 3D—three-dimensional; LVEF—left ventricular ejection fraction; *—statistically significant difference compared to baseline (*p* < 0.05).

Only one patient in the control group (4%) and none in the ivabradine group had a decrease in LVEF < 50%. The mean baseline LVEF was 63.5 ± 3.4% in the ivabradine and 64.4 ± 4.5% in the control group (*p* = 0.478). The lowest LVEF was observed at 6 months follow-up and was 61.2 ± 3.5% in the ivabradine group and 62.1 ± 4.9% in the controls (*p* = 0.493). No significant differences in LVEF at any time were noted between groups (Table 2). A statistically significant reduction in 2D LVEF from the baseline of 1.5% was observed after four cycles of AC and 2.3% at 6 months follow-up in the ivabradine group (*p* = 0.022 and *p* = 0.008) and 3.3% and 2.3% in the controls (*p* = 0.016 and 0.027). In contrast, a statistically significant decrease in 3D LVEF of 2.5% from the baseline after four cycles of AC and 2.7% at 6 months follow-up was noticed only in the control group (*p* = 0.029 and *p* = 0.038). Changes in LVEF during the study are shown in Figure 6.

Symptomatic CTRCD was diagnosed in one patient from the control group when HF symptoms appeared, and reduced LVEF (31%) was determined after four cycles of anthracyclines. After guideline-based HF therapy [26], LVEF recovered to 45%. 

#### 3.4.5. RV Dysfunction

**Figure 7:**
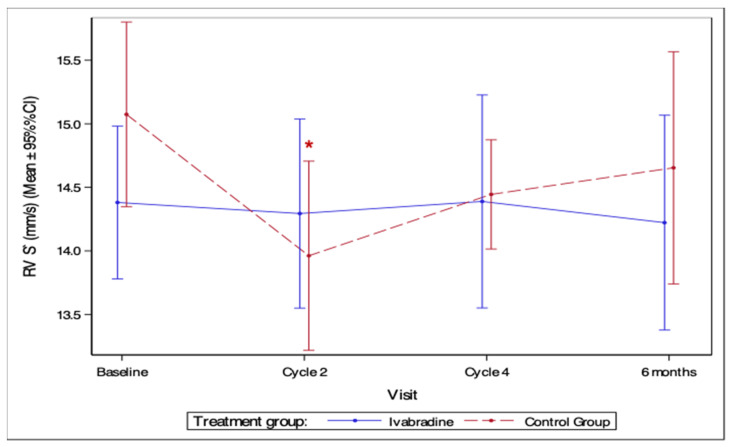
Measurements of right ventricle tricuspid annular systolic velocity during the study period. RV—right ventricle; S’—tricuspid annular systolic velocity by tissue Doppler; *—statistically significant difference compared to baseline (*p* < 0.05).

For RV dysfunction, no difference occurred across groups over the study period (*p* = 0.85). It was only in the control group that there was a significant reduction in S’ after two cycles of chemotherapy (*p* = 0.002) (Figure 7).

#### 3.4.6. Myocardial Work Indices

**Figure 8:**
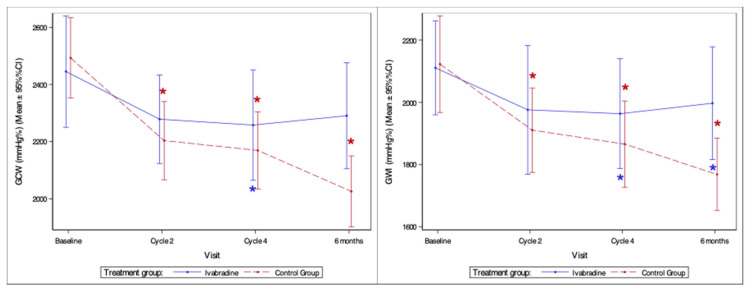
Change in global myocardial constructive work and global myocardial work index. GCW—global constructive work; GWI—global work index; *—statistically significant difference compared to baseline (*p* < 0.05).

Significant differences between groups were observed for GCW and GWI at 6 months follow-up (*p* = 0.014 and *p* = 0.025) (Figure 8). Greater constructive work and work index was preserved in the ivabradine group.

In the ivabradine group, a significant decrease in GCW was observed after four AC cycles (*p* = 0.008), whereas in the control group, GCW decreased with each visit (after two AC cycles *p* = 0.012, after four AC cycles *p* < 0.001, and at 6 months follow-up *p* < 0.001). A significant reduction in GWI was measured in the ivabradine group after four AC cycles and at 6 months follow-up (*p* = 0.015 and *p* = 0.042), while in the control group GWI decreased steadily at each visit (*p* = 0.017; *p* = 0.007 and *p* < 0.01, respectively) (Figure 8).

Table 2 shows all changes in echocardiographic parameters during the study.

#### 3.4.7. Mild Asymptomatic Cancer-Therapy-Related Cardiac Dysfunction

In summary, mild asymptomatic CTRCD, defined by the 2022 ESC Cardio-Oncology Guidelines, was detected in 29 patients (60.4%). Of these, 10 were in the ivabradine group and 19 in the control group (*p* = 0.045). 

The alteration of cardiac biomarkers is presented in Table 3.

According to the recent recommendations, patients who developed mild CTRCD were prescribed cardioprotective treatment with 2–4 mg of perindopril and monitored every 4 weeks until test results normalized.

**Figure 9:**
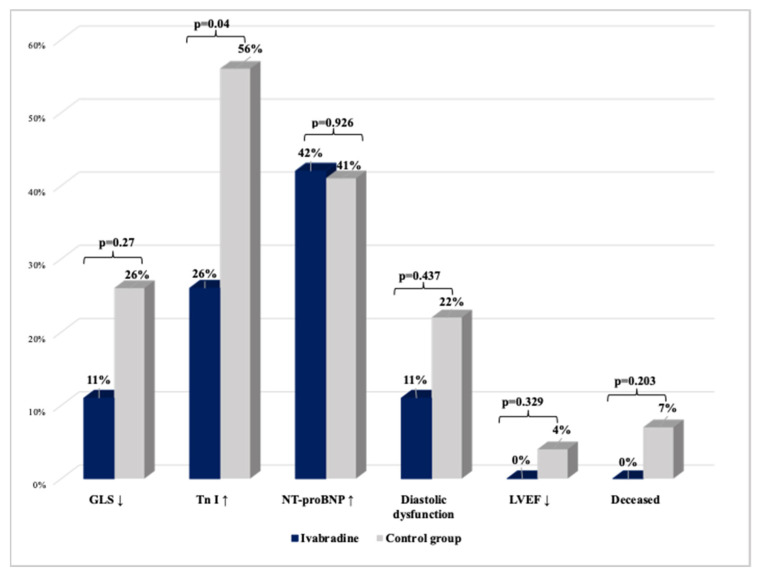
Myocardial damage markers. GLS—global longitudinal strain; LVEF—left ventricular ejection fraction; NT-proBNP—N-terminal pro-brain natriuretic peptide; Tn I—troponin I.

All myocardial damage markers are presented in Figure 9.

#### 3.4.8. Incidence of Adverse Effects of Ivabradine

The use of ivabradine was safe and well-tolerated. No patient had to stop treatment with ivabradine. An ECG was recorded for every patient at every visit, and no significant changes in PQ, QRS, or QTc intervals were detected. However, three patients reported visual side effects (phosphenes). All three patients reported Grade 1 visual side effects: 1–2 recurrent low-intensity flashing lights in their eyes, which did not interfere with their activities of daily living and resolved spontaneously after a few days. 

## 4. Discussion

We performed a prospective, randomised, open-label clinical trial to evaluate the use of ivabradine for the primary prevention of anthracycline-induced cardiotoxicity. 

Our study is the first to investigate ivabradine’s cardioprotective properties and provide a comprehensive analysis of changes in cardiac biomarkers and echocardiographic parameters during anthracycline-based chemotherapy. 

The cardioprotective effects of ivabradine may be explained by the pleiotropic effects. It reduces heart rate without affecting myocardial inotropic function, reducing oxygen demand and maintaining diastolic time. As a result, it reduces myocardial stress and may improve myocardial deformation and coronary microcirculation following anthracycline exposure [27,28,29,30]. In animal studies, ivabradine exerts anti-remodelling effects through multiple mechanisms, including antifibrotic, anti-inflammatory, antioxidant, and anti-apoptotic effects [13,14]. 

We compared our results with four major trials that examined the cardioprotective effects of ACEIs and BBs on anthracycline-induced cardiopathy: OVERCOME (“Prevention of left ventricular dysfunction with enalapril and carvedilol in patients submitted to intensive chemotherapy for the treatment of malignant hemopathies”) [31], PRADA (“Prevention of cardiac dysfunction during adjuvant breast cancer therapy”) [32], CECCY (“Carvedilol for the prevention of chemotherapy-related cardiotoxicity”) [24], and ICOS-ONE (“A multicentre randomised trial comparing two strategies for guiding prevention with enalapril: The International Cardio-Oncology Society-one trial”) [5]. The similarities and differences are listed below.

### 4.1. Reduction in the GLS during Cardiotoxic Chemotherapy

GLS is an echocardiographic measure of left ventricular function that is a more sensitive marker of subclinical myocardial dysfunction than LVEF [33,34]. Anthracycline therapy is associated with cardiomyocyte injury, loss of cardiac contractile function, inflammation, and the development of diffuse fibrosis, which may decrease GLS [35,36]. GLS decline in anthracycline-treated patients is an essential marker of subclinical myocardial dysfunction and may help to identify patients at higher risk of developing clinically significant cardiac events [37,38]. However, GLS has not been widely used in clinical trials to assess LV dysfunction. In addition, investigators have often used different rates of decline in GLS, making it difficult to compare data. The >15% reduction in GLS was observed in 19% of our patients compared to 22–31% in other studies [39,40]. Our GLS results are consistent with the randomised trial investigating the cardioprotective properties of ivabradine in anthracycline-treated breast cancer patients, which showed preservation of mean GLS with ivabradine [15]. Positive effects on GLS have been observed with candesartan treatment [41].

### 4.2. Increase in Tn I Levels

An increase in troponin levels was observed frequently in previous trials of cardioprotective therapy, which was not necessarily associated with early deterioration in LV function, and a clear dose-dependency of anthracyclines was observed. Amelioration of troponin elevation was reported in patients treated with carvedilol and metoprolol [24,41]. 

We found that anthracycline-containing chemotherapy increased troponin I, even when low-to-moderate doses of anthracyclines were used in patients with a low risk of cardiotoxicity. In our study, troponin elevation was found in almost 42% of patients and were most common after four cycles of chemotherapy. Previous studies have reported troponin elevations in approximately 25–30% of patients treated with anthracyclines [42,43]. Therefore, a slightly higher frequency of troponin elevation in our sample could be due to differences in assessment (we performed a high-sensitivity troponin I test in all patients) and more frequent testing. Ivabradine treatment significantly reduced the incidence of the anthracycline cardiotoxicity measured by troponin concentration.

### 4.3. Increase in Natriuretic Peptide Levels

The increase in natriuretic peptides was observed in all trials of cardioprotective treatment with ACEIs and BBs, with no effect of different therapies on its frequency [24,32]. Similarly, the elevation of natriuretic peptides did not differ between the intervention and control groups in our study.

### 4.4. Development of Diastolic Dysfunction

Diastolic dysfunction or deterioration may predispose the development of HF and is an early sign of cardiotoxic injury [44,45]. In a previous retrospective study of cardio-oncology patients, we showed that diastolic dysfunction worsens survival in cancer patients [46]. In the cardioprotective treatment trials, only treatment with carvedilol had a beneficial effect on diastolic function, up until now [24]. Ivabradine did not affect the frequency of diastolic dysfunction in our cohort. 

In our study, we diagnosed diastolic dysfunction in 17% of patients, compared with up to 40% in other studies of patients treated with anthracyclines [40]. This difference could be explained by the fact that the assessment of diastolic function is complex and depends on several changes in echocardiographic parameters, patient age, and CV risk factors. 

### 4.5. Deterioration of LV Systolic Function

Significant cancer-therapy-related cardiac dysfunction (defined as a 10% reduction in LV ejection fraction, with values less than 50%) is rare with modern anthracycline doses, with a 1–2% prevalence [47]. This may be explained by the lower doses of AC used and better management of patients at high risk of cardiotoxicity [48]. The exclusion of patients at high risk of cardiotoxicity in our trial due to the use of concomitant medications may explain the expected low prevalence of severe CTRCD. The low incidence of moderate to severe cardiotoxicity in cardioprotective treatment with ACEI and BB trials corresponds to our data. However, mild asymptomatic CTRCD was diagnosed in more than half of our patients (60%), almost twice as often as in patients from the CARDIOTOX registry (31.6%). We found that preventing anthracycline-induced cardiotoxicity with ivabradine was less likely to result in mild asymptomatic CTRCD; the incidence of a significant reduction in GLS was 40% lower, the incidence of troponin elevation was four times lower, and the incidence of NT-proBNP increase was 12% lower in ivabradine-treated patients compared with controls. Furthermore, cardiac performance was less impaired regarding myocardial work parameters.

### 4.6. Alterations in Myocardial Performance

Although, GLS is a more sensitive method of assessing LV systolic function than LVEF, it has the disadvantage of being dependent on LV loading conditions. Non-invasive assessment of myocardial work indices improves assessment of myocardial performance by incorporating load and strain in the analysis. Cardiotoxic cancer treatments adversely affect myocardial work indices, indicating a decline in cardiac function. The extent and timing of these changes may vary depending on the type and dose of cancer treatment and individual patient factors [20,49,50]. To the best of our knowledge, we were the first to investigate the effect of ivabradine on changes in MW indices. We observed a significant beneficial effect of ivabradine, particularly on GWI and GCW. Estimating myocardial work parameters can be considered as a potential endpoint for the cardio-oncology trials. 

Our data support previous evidence that early assessment of subclinical cardiotoxicity (using measurement of Tn I, NT-proBNP, GLS, and MW indices) in patients undergoing cardiotoxic cancer therapy may lead to early detection of LV dysfunction and timely initiation of appropriate management to prevent serious complications and improve survival in cancer patients.

We believe that the cardioprotective properties of ivabradine could be demonstrated in a larger population of cancer patients, including those at higher risk of cardiotoxicity.

## 5. Study Limitations

The main limitations of the trial were that it was a single-centre trial with a small number of patients. This study can be interpreted as a pilot study, and with a new sample size calculation based on the data collected, further research is warranted. 

Also, the study only included women with the highest prevalence of breast cancer. Therefore, the results cannot be generalized to larger populations. We planned to have patients of all genders with different types of cancer who were due to receive anthracycline treatment, but we could only include women with breast cancer. We think that the main reason is that most patients treated with anthracyclines are women with breast cancer.

Another limitation was that only patients with a low risk of cardiotoxicity could be included. However, enrolling patients at high risk of cardiotoxicity in cardioprotective trials is challenging because most are already being treated with ACEIs/ARBs and BBs, which are known to have beneficial cardioprotective effects. As the follow-up results may have been influenced by the cardioprotective treatment with ACEIs in CTRCD, we consider it unethical to withhold treatment in this case.

## 6. Conclusions

Ivabradine may protect female patients treated with anthracyclines, primarily for those with breast cancer and a higher heart rate from cardiotoxicity, characterized by a new rise in troponin levels and subclinical myocardial dysfunction diagnosed by myocardial work indices. More extensive multicentre trials are needed to provide statistically robust evidence.

## Figures and Tables

**Table 1:** The baseline characteristics of the patients.

Baseline Characteristics	Ivabradine *n =* 21 (%)	Controls *n* = 27 (%)	*p*-Value
Age, years (mean ± SD, range)	47.8 ± 9.9, 34–66.5	48 ± 10.2, 34–66.5	0.935
Cancer type, *n* (%)			0.449
Right breast	10 (47.6)	10 (37)	
Left breast	11 (52.4)	14 (51.9)	
Both breasts	0	1 (3.7)	
Sarcoma	0	2 (7.4)	
Cancer stage, *n* (%)			0.509
I	2 (9.5)	6 (22.2)	
II	14 (66.7)	15 (55.6)	
III	5 (23.8)	5 (18.5)	
IV	0	1 (3.7)	
Cancer grade, *n* (%)			0.309
1	0	0	
2	13 (65)	13 (50)	
3	7 (35)	13 (50)	
HER2, *n* (%)			0.333
Positive	7 (35)	6 (22.2)	
Negative	13 (65)	21 (77.8)	
BRCA, *n* (%)			0.928
Positive	3 (15.8)	4 (14.8)	
Negative	16 (84.2)	23 (85.2)	
Chemotherapy, *n* (%)			0.060
Adjuvant	10 (47.6)	20 (74.1)	
Neoadjuvant	11 (52.4)	7 (25.9)	
Anthracyclines, *n* (%)			0.545
Doxorubicin	15 (71.4)	21 (77.8)	
Epirubicin	6 (28.6)	6 (22.2)	
Cumulative anthracycline dose			0.425
Doxorubicin, mg/m^2^ (mean ± SD)	236 ± 70	246 ± 69	
Epirubicin, mg/m^2^ (mean ± SD)	360 ± 70	360 ± 70	
CV risk factors, *n* (%)			
Hypertension	3 (15)	2 (7.4)	0.404
Diabetes	0	0	
Dyslipidaemia	13 (65)	21 (77.8)	0.333
Smoking	1 (5)	6 (22.2)	0.101
Obesity (BMI > 30 kg/m^2^)	4 (19)	2 (7.4)	0.226
Kidney dysfunction (GFR < 60 mL/min./1.73 m^2^)	0	0	
Cardiotoxicity risk group, *n* (%)			0.519
Low	17 (81)	23 (85.2)	
Medium	4 (19)	3 (11.1)	
High	0	1 (3.7)	
Very high	0	0	
HF stage, *n* (%)			0.356
A	4 (19)	4 (14.8)	
B	4 (19)	8 (29.6)	
C	0	1 (3.7)	
D	0	0	
Anaemia (Hb < 117 g/L), *n* (%)	12 (60)	15 (55.6)	0.761
CRP > 5 mg/L, *n* (%)	2 (9.5)	5 (18.5)	0.381
Vitamin D < 75 nmol/L, *n* (%)	12 (70.6)	19 (86.4)	0.226
Myocardial damage markers at baseline, *n* (%)			
Tn I > 16 ng/L	0	0	
NT-proBNP > 125 ng/L	3 (14.3)	9 (33.3)	0.131
GLS > −18%	0	1 (3.7)	0.373
LVEF < 55%	0	0	
Diastolic LV dysfunction	1 (4.8)	2 (7.4)	0.707
Echocardiographic parameters at baseline, *n* (%)			
LAVI > 34 mL/m^2^	5 (23.8)	5 (18.5)	0.654
Transmitral E velocity > 50 cm/s	7 (33.3)	10 (37)	0.790
Transmitral A velocity < 60 to < 120 cm/s	12 (57.1)	11 (40.7)	0.259
Transmitral E/A ratio < 0.8 to > 2.0	7 (33.3)	6 (22.2)	0.390
Mitral E/e’ ratio > 14	0	0	
e’ med. < 7 cm/s	1 (4.8)	4 (14.8)	0.258
e’ lat. < 10 cm/s	2 (9.5)	2 (7.4)	0.792
IVRT < 70 to > 100 msec	12 (57.1)	8 (29.6)	0.037
Transmitral E velocity DT < 160 to > 220 msec	11 (52.4)	9 (33.3)	0.184
RV S’ < 9.5 cm/s	0	0	
TAPSE < 17 cm	0	0	
2D LVEF < 55%	0	0	
GWI < 1310 mmHg%	0	0	
GCW < 1543 mmHg%	0	0	
GWW > 287 mmHg%	0	0	
GWE < 90%	1 (5.3)	0	0.376
3D LVEF< 55% *	0	0	

2D—two-dimensional; 3D—three-dimensional; BMI—body mass index; BRCA—breast cancer gene 1; DT—deceleration time; GCW—global constructive work; GFR—glomerular filtration rate; GLS—global longitudinal strain; GWE—global work efficiency; GWI—global work index; GWW—global wasted work; Hb—haemoglobin; HER2—human epidermal growth factor receptor 2; HF—heart failure; CRP—C-reactive protein; IVRT—isovolumic relaxation time; LAVI—left atrial volume index; LV—left ventricle; LVEF—left ventricular ejection fraction; NT-proBNP—N-terminal natriuretic pro-peptide; RV—right ventricle; RV S’—tricuspid annular systolic velocity by tissue Doppler; SD—standard deviation; TAPSE—tricuspid annular plane systolic excursion; Tn I—troponin I. * 3D echocardiography was performed in 32 patients (14 in ivabradine and 18 in the control group).

**Table 2:** Changes in echocardiographic parameters between groups.

Parameter	All (*n* = 48)	Ivabradine (*n* = 21)	Controls (*n* = 27)	*p*-Value
Baseline				
2D LVEF (mean ± SD)	64 ± 4.0	63.5 ± 3.4	64.4 ± 4.5	0.478
3D LVEF (mean ± SD)	64.3 ± 4.1	62.7 ± 4.0	65.5 ± 3.8	0.055
RV function				
TAPSE (mean ± SD)	22 ± 2.7	22.2 ± 3.4	21.8 ± 2.2	0.661
RV S’ (mean ± SD)	14.8 ± 1.6	14.4 ± 1.3	15.07 ± 1.8	0.152
Myocardial work indices				
GWI (median; Q1–Q3; range)	2055.5 (1895–2288; 1552–2904)	2090 (1911–2341; 1552–2697)	2021 (1846–2288; 1571–2904)	0.820
GCW (mean ± SD)	2471.3 ± 359.2	2445.2 ± 404.4	2492.8 ± 325	0.674
GWW (median; Q1–Q3; range)	98 (75–131; 35–263)	82 (63–144; 35–263)	105 (77–131; 38–156)	0.622
GWE (median; Q1–Q3; range)	95 (95–97; 86–98)	96 (94–97; 86–98)	95 (95–97; 93–98)	0.826
After 2 anthracyclines cycles				
2D LVEF (mean ± SD)	63.3 ± 4.0	62.1 ± 3.4	64.0 ± 4.3	0.129
3D LVEF (mean ± SD)	63.6 ± 2.6	63.5 ± 2.3	63.8 ± 3.0	0.751
RV function				
TAPSE (mean ± SD)	21.5 ± 2.9	22 ± 3.1	21.1 ± 2.7	0.327
RV S’ (mean ± SD)	14.1 ± 1.7	14.3 ± 1.4	14.0 ± 1.8 *	0.534
Myocardial work indices				
GWI (median; Q1–Q3; range)	1911.5 (1671–2229.5; 1043–2587)	1959 (1766–2253; 1043–2587)	1826 (1632–2206; 1416–2470) *	0.412
GCW (mean ± SD)	2235.3 (308.1)	2278.5 (300.8)	2203.4 (316.2) *	0.453
GWW (median; Q1–Q3; range)	84 (68.5–108; 27–438)	81 (48–125; 35–438)	86 (70–106; 27–226)	1.000
GWE (median; Q1–Q3; range)	96 (95–96.5; 84–98)	96 (95–97; 84–98)	95 (95–96; 90–98)	0.348
After 4 anthracyclines cycles				
2D LVEF (mean ± SD)	61.4 ± 5.492	62 ± 3.1 *	61.1 ± 6.7 *	0.534
3D LVEF (mean ± SD)	63.0 ± 3.8	63.1 ± 4.3	63 ± 3.3 *	0.964
RV function				
TAPSE (mean ± SD)	21.3 ± 2.9	22.2 ± 2.5	20.7 ± 3.0	0.087
RV S’ (mean ± SD)	14.4 ± 1.3	14.4 ± 1.7	14.4 ± 1.1	0.902
Myocardial work indices				
GWI (median; Q1–Q3; range)	1906.5 (1747.5–2111.5; 684–2518)	1946 (1616–2240; 1468–2518) *	1890 (1769–2073; 684–2453) *	0.591
GCW (mean ± SD)	2205.5 ± 356.1	2257.9 ± 388.7 *	2169.3 ± 334.7 *	0.423
GWW (median; Q1–Q3; range)	89 (59–114; 23–150)	76.5 (61–107; 32–149)	96 (58–121; 23–150)	0.424
GWE (median; Q1–Q3; range)	96 (95–97; 84–98)	96 (95–97; 94–98)	95 (94–97; 84–98)	0.113
At 6 moths follow-up				
2D LVEF (mean ± SD)	61.8 ± 4.4	61.2 ± 3.5 *	62.1 ± 4.9 *	0.493
3D LVEF (mean ± SD)	62.3 ± 4.7	61.7 ± 5.1	62.8 ± 4.4 *	0.512
RV function				
TAPSE (mean ± SD)	21.3 ± 2.7	21.3 (2.3)	21.3 ± 3	0.972
RV S’ (mean ± SD)	14.5 ± 2.04	14.2 (1.7)	14.6 ± 2.3	0.497
Myocardial work indices				
GWI (median; Q1–Q3; range)	1839.5 (1637–2084.5; 1055–2677)	1979.5 (1665–2339; 1396–2677) *	1795 (1632–2009; 1055–2171) *	0.044
GCW (mean ± SD)	2134.1 ± 357.0	2290.6 ± 373.1	2025.8 ± 307.8 *	0.014
GWW (median; Q1–Q3; range)	100.5 (70–142; 29–266)	104.5 (75–150; 42–260)	96.5 (65–132; 29–266)	0.676
GWE (median; Q1–Q3; range)	95 (92.5–96; 87–98)	95 (94–96; 90–98)	95 (92–96; 87–98)	0.709

2D—two-dimensional; 3D—three-dimensional; GCW—global constructive work; GFR—glomerular filtration rate; GLS—global longitudinal strain; GWE—global work efficiency; GWI—global work index; GWW—global wasted work; LVEF—left ventricular ejection fraction; RV—right ventricle; RV S’—tricuspid annular systolic velocity by tissue Doppler; SD—standard deviation; TAPSE—tricuspid annular plane systolic excursion; *—statistically significant difference compared to baseline (*p* < 0.05).

**Table 3:** Changes in cardiac biomarkers.

Parameters	All (*n* = 48)	Ivabradine (*n* = 21)	Controls (*n* = 27)	*p*-Value
Baseline				
Tn I (median; Q1–Q3; range)	1; 0–1.5; 0–5	1; 0–1; 0–3	1; 0–2; 0–5	0.465
NT-proBNP (median; Q1–Q3; range)	70.8 (59.2–128.3; 21–846.6)	68.5 (60.3–98; 21–190.5)	74.8 (58.3–152; 47–846.6)	0.418
After 2 anthracyclines cycles				
Tn I (median; Q1–Q3; range)	3 (2–5; 0–172)	3 (2–4; 1–9) *	3 (2–6; 0–172) *	0.579
NT-proBNP (median; Q1–Q3; range)	82.1 (55.3–130.6; 26.6–945)	84 (52.4–138; 32–281.6)	80.2 (65.7–116.4; 26.6–945)	0.918
After 4 anthracyclines cycles				
Tn I (median; Q1–Q3; range)	12 (7–24; 2–73)	10 (6–21; 2–73) *	14 (9–31; 2–70) *	0.444
NT-proBNP (median; Q1–Q3; range)	84 (49.3–148; 26.9–16,048.7)	80 (53.4–153.1; 26.9–233.4)	84 (44–148; 29.5–16,048.7)	0.908
At 6 months follow-up				
Tn I (median; Q1–Q3; range)	5.5 (3–13; 1–109)	4 (3–7; 1–42) *	7.5 (3–20; 2–109) *	0.124
NT-proBNP (median; Q1–Q3; range)	84.4 (51.2–150.4; 20–10,032.7)	71.8 (49.7–131.1; 29.8–504.9)	86.4 (57.2–163.8; 20–10,032.7)	0.334

AC—anthracyclines; NT-proBNP—N-terminal pro-brain natriuretic peptide, Tn I—troponin I. *—statistically significant difference compared to baseline (*p* < 0.05).

## Data Availability

The data presented in this study are available on request from the corresponding author. The data are not publicly available due to privacy. The study investigator, Eglė Čiburienė, had full access to all of the data in the study and takes responsibility for the integrity of the data and the accuracy of the data analysis.
